# Generation of a Small Library of Natural Products Designed to Cover Chemical Space Inexpensively

**DOI:** 10.20900/pf20190005

**Published:** 2019-08-09

**Authors:** Steve O’Hagan, Douglas B. Kell

**Affiliations:** 1 School of Chemistry, The University of Manchester, 131 Princess St, Manchester M1 7DN, UK; 2 Manchester Institute of Biotechnology, The University of Manchester, 131 Princess St, Manchester M1 7DN, UK

**Keywords:** drug transporters, cheminformatics, endogenites, metabolomics, clustering

## Abstract

Natural products space includes at least 200,000 compounds and the structures of most of these compounds are available in digital format. Previous analyses showed (i) that although they were capable of taking up synthetic pharmaceutical drugs, such exogenous molecules were likely the chief ‘natural’ substrates in the evolution of the transporters used to gain cellular entry by pharmaceutical drugs, and (ii) that a relatively simple but rapid clustering algorithm could produce clusters from which individual elements might serve to form a representative library covering natural products space. This exploited the fact that the larger clusters were likely to be formed early in evolution (and hence to have been accompanied by suitable transporters), so that very small clusters, including singletons, could be ignored. In the latter work, we assumed that the molecule chosen might be that in the middle of the cluster. However, this ignored two other criteria, namely the commercial availability and the financial cost of the individual elements of these clusters. We here develop a small representative library in which we to seek to satisfy the somewhat competing criteria of coverage (‘representativeness’), availability and cost. It is intended that the library chosen might serve as a testbed of molecules that may or may not be substrates for known or orphan drug transporters. A supplementary spreadsheet provides details, and their availability via a particular supplier.

## Introduction

It is by now evident (e.g., [1–29]) that pharmaceutical drugs exploit endogenous transporters that normally transport biological metabolites (whether they are endogenous, or are represented by exogenous natural products). The possibly surprising quantitative consequence of these and other studies is that diffusion of such drugs through the phospholipid bilayer portions of undamaged biological membranes is in fact negligible [1,3,5–7,10,11,13,30].

The principle of molecular similarity (e.g., [31]) implies that small molecules with similar structures will bind to the same kinds of proteins and exhibit similar kinds of activity. We [2,16,32–37] and others (e.g., [38–43]) have thus sought to assess the extent to which marketed drugs are similar in structural terms to endogenous human metabolites (that we sometimes refer to as “endogenites”). The criterion of being marketed was used because this implied that the drugs were efficacious and (since almost all were to be taken orally and/or required to interact with intracellular targets) capable of being transported across at least one biological membrane. It turned out [36] that when standard encodings were employed, and a Tanimoto similarity exceeding ~0.8 was used as a criterion of “similarity”, all drugs could be seen to be similar to either endogenites (~15%) or (more frequently) to natural products (commonly of plant and microbial origin), but that for similarities below this the various encodings often gave completely different rank orders.

This latter finding, the importance of natural products in the natural selection of transporters, raises a more ecological kind of thinking [44–46], in which it becomes obvious that the ability to take up natural products (such as cocaine [47], ergothioneine [48,49], and many others) is indeed likely to improve the fitness of an organism with a protein transporter capable of transporting them.

As with the products of many other genes uncovered by the systematic genomic sequencing programmes (e.g., [50]), many transporters remain “orphans” [12], with no known substrates. Clearly one strategy to “de-orphanise” them would be to try all kinds of substrates in parallel and use the methods of ‘untargeted metabolomics’ to assess their uptake differentially in cells expressing different amounts of the transporter of interest (e.g., [48]). Another method is to try many drugs serially, but this would be prohibitively expensive for large libraries. Consequently one strategy (e.g., [37,51]) that we have chosen is to develop a small and ‘representative’ library that might reasonably cover natural products space efficiently and inexpensively, and that might then be used to assess which of its members were substrates for particular transporters. Having established the greatest activities, those small molecule structures could then be used as “seeds” for the acquisition and analysis of other molecules with which to establish a suitable QSAR. Armed with that, and the concentrations of the transporters themselves, one would then have the information necessary to permit the calculation of the activity of that transporter for any drugs in different cells.

The only “missing piece” in the generation of this kind of library hinged on the commercial availability and cost of the molecules themselves. As with other programs of this type (e.g., [52–56]), the desire is for a library that is both diverse yet accessible. In collaboration with a commercial partner, we have now developed a library that is at once small, suitably comprehensive, and with a price that is accessible to most reasonably funded laboratories. It is this that we here describe.

## Materials and Methods

As in our related projects (e.g., [15,32–35]), we developed and ran our cheminformatics routines in the KNIME environment [57,58], including on occasion two nodes available from the Molport website at https://www.molport.com/shop/knime-nodes. We made considerable use of the RDKit package [59], especially the most recent “patterned” fingerprint encoding. Other software used is referenced in the Results section.

## Results

Our previous work [37] separated the large UNPD (Universal Natural Products Database [60] http://pkuxxj.pku.edu.cn/UNPD/)(and the commercial Dictionary of Natural Products (DNP) library) of natural products into appropriate clusters, ranked by cluster size. To create a small and suitably priced library that might nonetheless give good coverage of it, we used the following general algorithm (given as pseudocode):

- Rank each cluster according to its size
- Filter out duplicate molecules
- Pick a subset of each cluster proportional to the square root of the cluster size and such that the total number of subset compounds selected over all clusters is equal to some maximum library size (we initially chose 1920)
- Pick the molecules within the MolPort database closest to each of the cluster subset members
- Continue for any cluster subset containing more than five molecules, stop when no further cluster subsets pass these criteria

**Figure 1:**
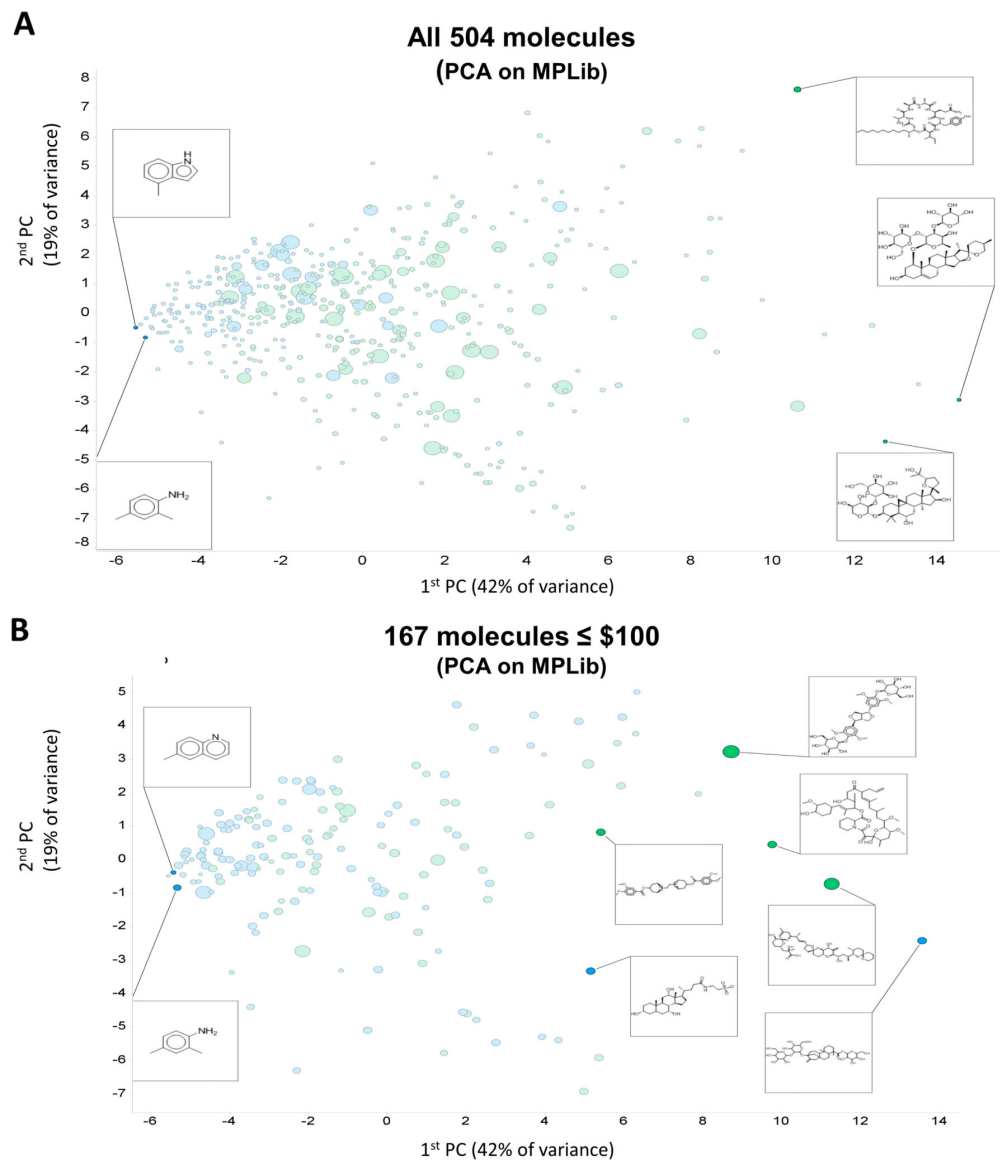
Initial coverage of natural products space as described in the text. Exact matches to cluster centres (blue) or nearby isomers (green) available in the Molport collection are labelled accordingly. Price is encoded via symbol size from $10 to $5713. (**A**) Full set of 504 molecules. (**B**) Reduced set of 167 molecules costing $100 or less.

Figure 1A shows a PCA plot of 504 molecules that met these criteria. Clearly some molecules are very expensive and fail our criterion of affordability. We also show five representative structures, indicating a variation in complexity over the first PC. Exact matches between Molport molecules and those in the databases are also more common towards the left-hand side of the first PC.

Figure 1B shows the same data when they were subject to a price ceiling of $100 per molecule (regardless of quantity). We then added two more criteria.

- Filter out any molecules with SLogP > 5.0
- Keep only molecules that pass chosen price and availability criteria (usually this was at least 25 mg for less than $100)

The final filtered list of 167 library compounds, taken either at or near 167 unique cluster centres out of the total of 7363 clusters represents 2.27% of clusters. Taking cluster membership into account, these 167 clusters represent approx. 8200 compounds out of a total of 195,000 compounds (~4.2%). Whilst these figures seem small, they give no clear indication of how well the library covers natural product chemical space because most clusters are in fact tiny.

For purposes of visualization, we extracted a random subset of 5000 molecules from the UNPD dataset studied previously [37]. We used the full set of RDKit’s numerical scalar descriptors, except that correlated descriptors were filtered out with a correlation threshold of 0.98, and z-score normalized (descriptors as available in KNIME were used, see https://www.rdkit.org/docs/GettingStartedInPython.html#list-of-available-descriptors).

**Figure 2:**
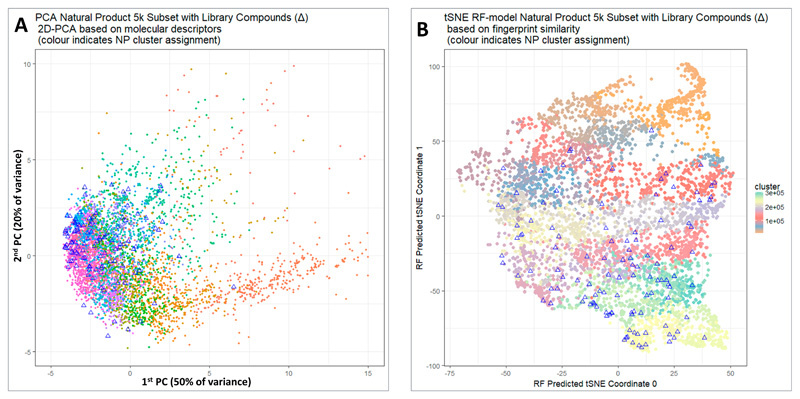
(**A**) Visualisation of the coverage of natural product(-like) space when molecules are selected from individual clusters. Principal components analysis was performed after normalizing to unit variance using a standard KNIME workflow. 5000 molecules are shown for purposes of visualization, and the 118 molecules closest to cluster centres that fulfilled our other criteria are indicated with triangles. (**B**) A t-SNE plot of the same data as in Figure 2A.

Figure 2A shows a Principal Components Analysis of those 5000 molecules (dots) together with the 117 molecules chosen (triangles) with filter criteria on SLogP, price and availability. It is clear that apart from the more sparsely populated part of chemical space to the right we do indeed have good coverage of the whole natural products (and natural-product-like) space. A more principled way of performing and visualizing dimensionality reduction is represented by the now-well-known variant of Stochastic Neighbor Embedding known as t-SNE [61]. In contrast to PCA, t-SNE is a nonlinear algorithm that does not admit projection of new data. To get round this, we first calculated the t-SNE coordinates in the normal way; we used Python Scikit-learn TSNE with default parameters and pre-computed distance matrix whose elements were (1.0—RDKit Pattern Fingerprint Tanimoto similarity) with t-SNE parameters: n_components = 2, perplexity = 30.0, early_exaggeration = 12.0, learning_rate = 200.0, n_iter = 1000, n_iter_without_progress = 300, min_grad_norm = 1e–07, metric = “precomputed”, init = “random”, method = “barnes_hut”, angle = 0.5). We trained a random forest model [62] using RDKit Pattern fingerprints as the input and the two t-SNE values as the output. We could then project in the new compounds of interest (cluster representatives) by passing them through the trained RF model in the same way. Thus Figure 2B shows a t-SNE plot of the same data, indicating that indeed this library covers the great majority of the chemical space. Those parts least covered (in orange) were not in fact from clusters that had only a very few members (and thus unable to provide sufficient members for a sensible QSAR analysis), but mainly from clusters containing compounds that did not meet our price or availability criteria.

96% of library compounds were exact matches to their target (TS = 1.0), most of the rest were either close isomers, tautomers or alternate charge states. The worst Tanimoto similarity between target and library compound found was 0.858 for the charged and non-charged versions of Chlorin e6.

Because of issues related to the same compound being represented by different tautomeric forms and charge states, *etc.*, we have not been able to find a foolproof procedure to standardize compound representations into a truly ‘canonical’ form, hence Tanimoto similarities somewhat less than 1.0 can nevertheless correspond to identical molecules.

**Figure 3:**
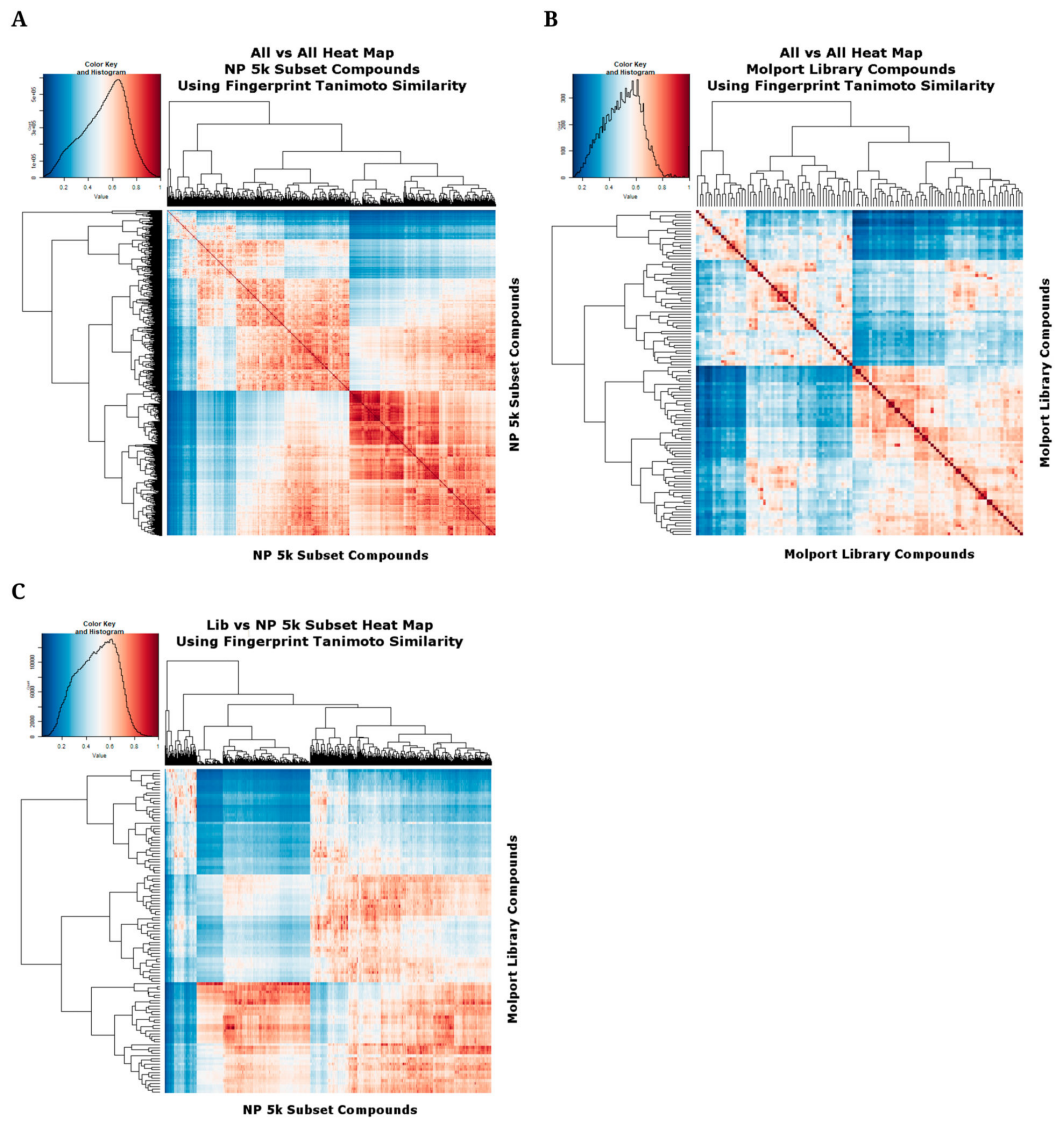
Heat map analyses of (**A**) The 5000-molecule subset and (**B**) The 117-molecule subset, based on their Tanimoto similarities. The analyses used the same workflows as those described in [32]. (**C**) The 5000-molecule subset versus the 117-molecule subset, based on their Tanimoto similarities. The analyses used the same workflows as those described in [32].

To assess the extent to which our clustering and subsetting has provided a much more widely separated set of molecules, we again encoded the molecules using the RDKit Pattern fingerprint. Figure 3A shows a heat map [63] of the 5000 subsample molecules as judged by their Tanimoto similarities, with a mode value being around 0.7. Figure 3B shows a similar analysis for the cluster representatives in the Molport library, where it is clear that far fewer have a mutual Tanimoto similarity exceeding 0.8, *i.e.*, we have covered the available space much more sparsely, as intended. Figure 3C shows the heat map for library samples *vs.* the 5000 subsample.

**Figure 4:**
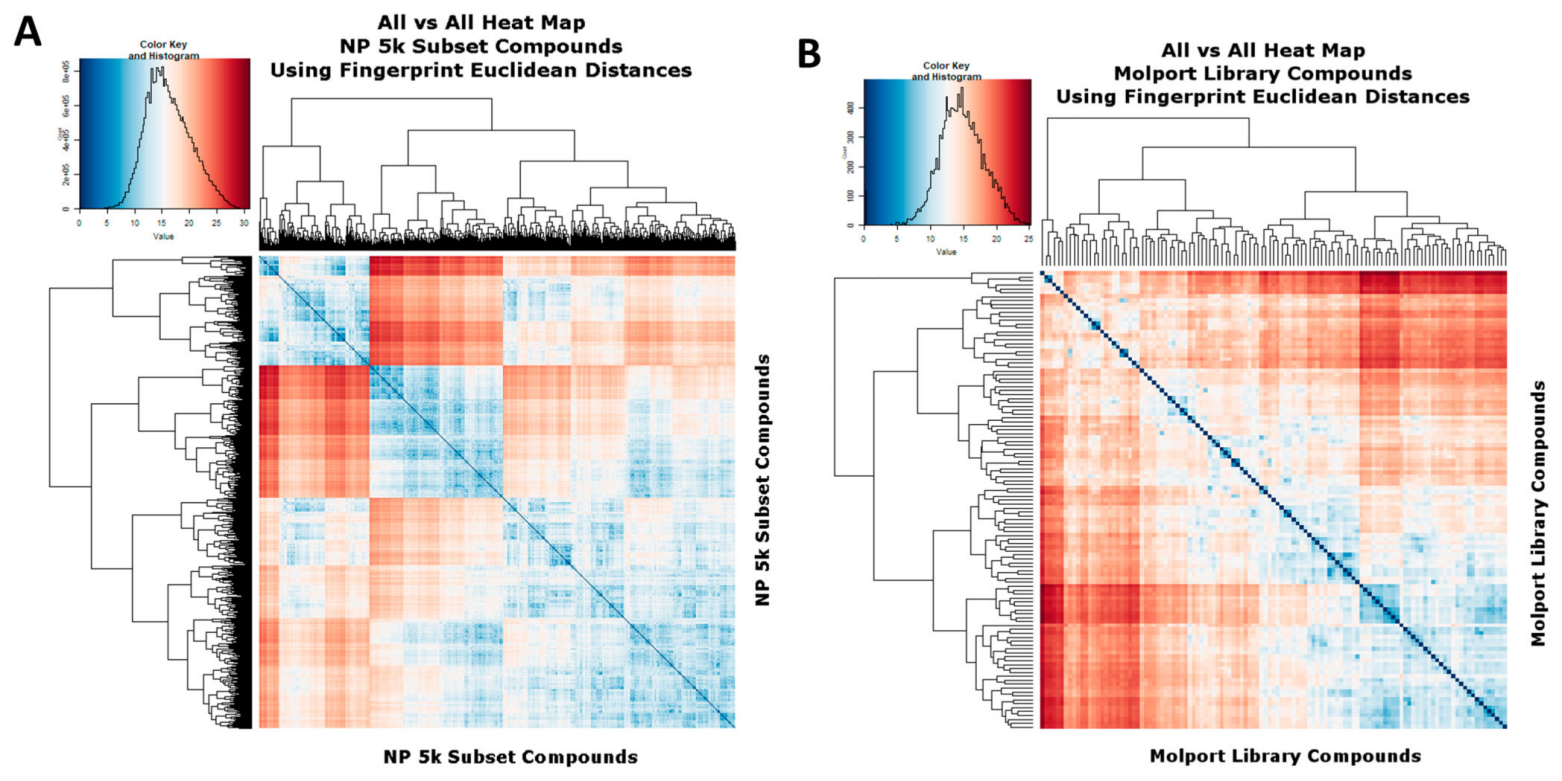
Heat map analyses of (**A**) The 5000-molecule subset, based on their Euclidean distances, as described in the text. Analyses and displays were otherwise as per Figures 2 and 3. (**B**) The 117-molecule subset, based on their Euclidean distances, as described in the text. Analyses and displays were otherwise as per Figures 2 and 3.

In a similar vein, Figure 4A,B show the similarities to each other of the 5k and cluster representatives when the fingerprint Euclidean distances (rather than Tanimoto similarities) are used. In this case the abscissa represents the square root of the number of different bits and blue represents more similar. Again the extraction of cluster representatives has pulled the average similarities away from each other.

**Figure 5:**
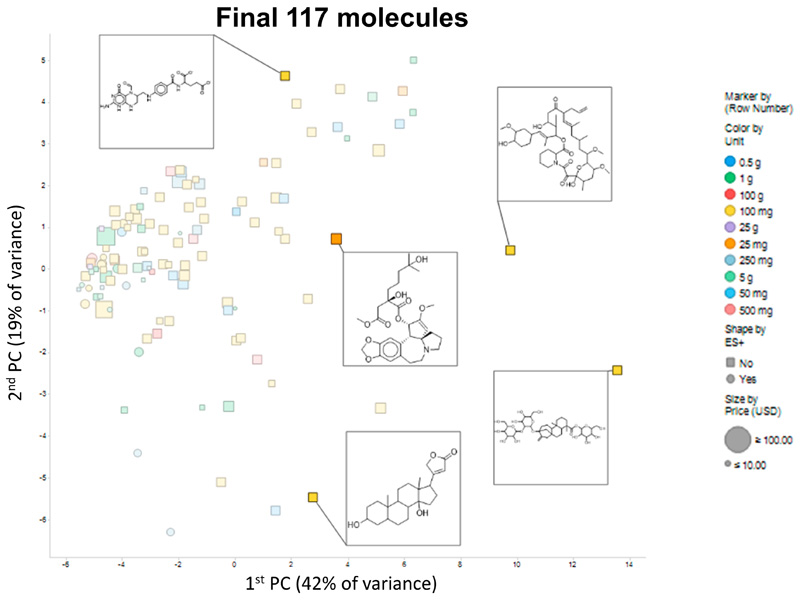
PCA plot of the 117-molecule subset, showing 5 representative molecules.

Finally, Figure 5 shows a PCA plot of the 117 molecules that passed our criteria; the amounts are encoded by colour and the cost by size, while the shape encodes whether their partial charge at neutral pH ≥ 0.5 and thus whether they are likely to be observed in positive ionization mode in a mass spectrometer. Again, notwithstanding some outliers to the right, there is a reasonable coverage of the available chemical space. The set of molecules is given in the supplementary spreadsheet. In practice, molecules go in and out of availability, and at the time of finalizing this manuscript only 116 of the 117 were in fact available. Consequently, we have not extended our analyses beyond this.

For those with larger budgets, we have also listed other representative quantities and guide prices in different tabs in the attached Supplementary Excel sheet. Both guide prices were optimized considering the total cost of compounds and shipping combinations.

## Discussion

Our aim in the present work, as part of a programme aimed at deorphanising (*i.e.*, finding the substrates for) membrane transporters, was to build on the recognition that many evolved and were selected to take up (or to efflux, or both) exogenous natural products (e.g., [36,48]). Although natural products space is occupied by far fewer known molecules (e.g., [60,64–68]) than either those possible [69] or the set of ~230 million mainly synthetic molecules collated e.g., at ZINC [70](http://zinc.docking.org/), it is still very large. Purchasing every possible molecule is prohibitively expensive, even for the subset of known (~200,000) natural products, and even if it were not many are either commercially unavailable or just singletons unsuited to our purposes (which aims to build a QSAR based on an initial hit followed by possible candidates that bear at least some chemical similarity to it). This is a simple extrapolation of the principle of molecular similarity, and the finding that molecules close in structure to a molecule with a certain activity are substantially enriched for that activity. In the landscape metaphor (e.g., [71,72]), this is equivalent to the assumption that a “starting” hit should at least be in the foothills of a more or less isolated mountain range that one would wish to explore (noting that in phenotypic sceening the objective function may involve or even favour polypharmacology).

A standard activity in cheminformatics is thus the production of reduced chemical libraries that cover the chemical space of interest [51], and that should still contain molecules that are (i) commercially available, and (ii) reasonably cheap. Cost provides a particularly clear filter [73]. Obviously this latter is a function of a laboratory’s budget, so we focused on the smallest library of this type that one might purchase in reasonable quantities for a somewhat arbitrary $5000 or so.

Plate-based screens are well known to be rather prone to edge effects [74,75], so while one might have suggested that we specify a number of molecules that might have a multiple of 90 wells or so (to allow for controls), we do not feel bound by this as numbers such as 117 allow arraying in a manner that easily avoids them.

## Conclusions

Conventional cheminformatics based on a prior cluster analysis of natural products space has allowed us to provide a set of small and relatively inexpensive libraries that may be useful in drug discovery and other assays (such as those seeking the substrates of orphan transporters).

## Supplementary Material

The supplementary materials listing molecules discussed in the text are available online at https://doi.org/10.20900/pf20190005.

Supplementary File 1: Supplementary Information
